# KIF4A facilitates cell proliferation via induction of p21-mediated cell cycle progression and promotes metastasis in colorectal cancer

**DOI:** 10.1038/s41419-018-0550-9

**Published:** 2018-04-30

**Authors:** Ping-Fu Hou, Tao Jiang, Fang Chen, Pei-Cong Shi, Hai-Qing Li, Jin Bai, Jun Song

**Affiliations:** 1 Cancer Institute, Xuzhou Medical University, Jiangsu 221002 Xuzhou, China; 2 Jiangsu Center for the Collaboration and Innovation of Cancer Biotherapy, Cancer Institute, Xuzhou Medical University, Jiangsu 221002 Xuzhou, China; 3 Department of General Surgery, The Affiliated Hospital of Xuzhou Medical University, Jiangsu 221002 Xuzhou, China

## Abstract

Kinesin family member 4A (KIF4A) was found to be implicated in the regulation of chromosome condensation and segregation during mitotic cell division, which is essential for eukaryotic cell proliferation. However, little is known about the role of KIF4A in colorectal carcinoma (CRC). This study explored the biological function of KIF4A in CRC progression and investigated the potential molecular mechanisms involved. Here, we found that KIF4A was remarkably upregulated in primary CRC tissues and cell lines compared with paired non-cancerous tissues and normal colorectal epithelium. Elevated expression of KIF4A in CRC tissues was significantly correlated with clinicopathological characteristics in patients as well as with shorter overall and disease-free cumulative survival. Multivariate Cox regression analysis revealed that KIF4A was an independent prognostic factor for poor survival in human CRC patients. Functional assays, including a CCK-8 cell proliferation assay, colony formation analysis, cancer xenografts in nude mice, cell cycle and apoptosis analysis, indicated that KIF4A obviously enhanced cell proliferation by promoting cell cycle progression in vitro and in vivo. Furthermore, gene set enrichment analysis, Luciferase reporter assays, and ChIP assays revealed that KIF4A facilitates cell proliferation via regulating the p21 promoter, whereas KIF4A had no effect on cell apoptosis. In addition, Transwell analysis indicated that KIF4A promotes migration and invasion in CRC. Taken together, these findings not only demonstrate that KIF4A contributes to CRC proliferation via modulation of p21-mediated cell cycle progression but also suggest the potential value of KIF4A as a clinical prognostic marker and target for molecular treatments.

## Introduction

Colorectal carcinoma (CRC) remains one of the most common malignancies and leading causes of cancer-related death worldwide^1^. In the past two decades, despite the dramatic improvements in the outcomes of CRC patients resulting from early diagnosis, the discovery of novel molecular targeted drugs, the development of neoadjuvant therapy and radical surgery advances, the 5-year overall survival (OS) of CRC patients remains unsatisfactory^2,3^. Therefore, it is essential to discover novel biological markers involved in the progression of CRC that can assist doctors in improving previous diagnostic practices and developing new therapeutic strategies for CRC patients.

Carcinogenesis is known to be a multistep process in which the loss of genomic stability accelerates the progression of colorectal cancer by facilitating the acquisition of multiple tumor-associated mutations^4^. The kinesin superfamily proteins (KIFs), classified into 14 subfamilies^5^, are microtubule (MT)-based motor proteins containing a conserved motor catalytic domain that binds to and hydrolyzes ATP to produce energy engaged in the transportation of a variety of cytoplasmic cargos and the regulation of MT stability^6^. Members of the kinesin superfamily play a key role in cell division, particularly for different stages of mitosis and cytokinesis, which can regulate the formation, orientation, and elongation of the mitotic spindle and the segregation of chromosomes in mitosis^7^.

One of the KIFs, kinesin family member 4A (KIF4A), an essential chromosome-associated molecular motor, maps to Xq13.1 in the human genome and encodes a 140-kDa protein that is composed of 1232 amino acids^8^ and is dominantly localized in the nucleus^9^. Previous studies have reported that KIF4A is involved in multiple significant cellular processes, especially in the regulation of chromosome condensation and segregation during mitotic cell division^10^, and dysregulation of KIF4A is considered to be involved in the DNA damage response^11^, abnormal spindle separation, and aneuploidy of daughter cells^12^, which further produces abnormal distribution of genetic materials. Notably, cells affected by aneuploidy are characterized by the loss of genetic stability, which is intensely suspected to be associated with tumorigenesis^13^. Previous studies have also demonstrated that KIF4A functions as an oncogene and plays critical roles in several malignancies, such as lung cancer, oral cancer^14^, breast cancer^15^, cervical carcinoma^16^, and hepatocellular carcinoma^17^. Nevertheless, the expression profile and the function of KIF4A in CRC remain unknown.

In the present study, to evaluate the role of KIF4A in CRC, we used a tissue microarray (TMA) along with retrospective CRC patient cohorts to investigate the relationship between KIF4A protein expression and clinicopathological features in CRC. In addition, we evaluated whether KIF4A could serve as an independent prognostic biomarker to target therapy for CRC patients. We demonstrated that KIF4A facilitates the proliferation of CRC in vitro and in vivo via transcriptionally regulating p21. Furthermore, KIF4A promotes metastasis in CRC cells. This study is the first to report the effect of KIF4A on cell proliferation and metastasis in CRC and to explain the molecular mechanism of KIF4A in CRC proliferation. These data provide new insights into the mechanisms of CRC tumorigenesis and support the potential value of KIF4A as a therapeutic target in CRC treatment.

## Results

### KIF4A is frequently upregulated in CRC tissues and cell lines

**Fig. 1:**
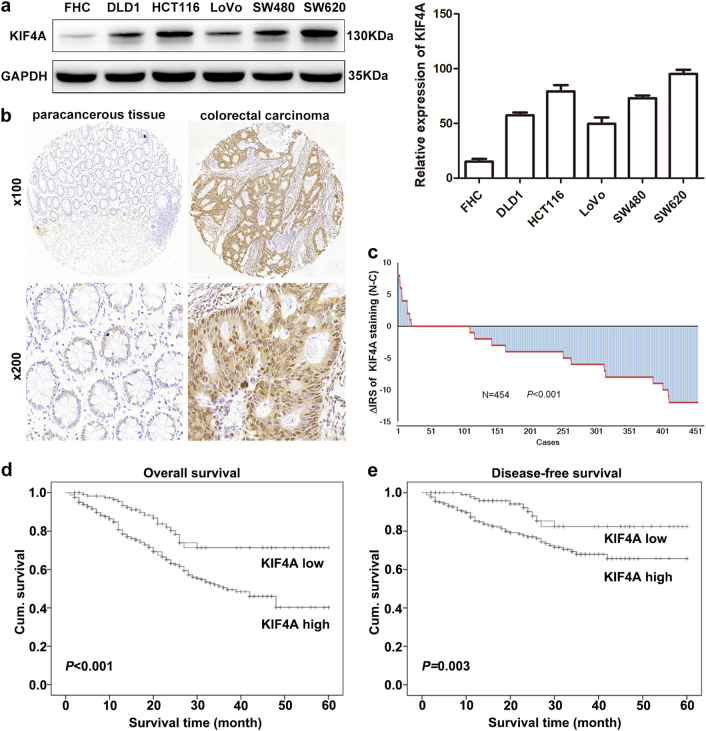
Expression of KIF4A is upregulated in CRC and negatively associated with overall and disease-free survival in CRC. **a** The expression level of KIF4A was detected by western blotting in five CRC cell lines and a normal colon epithelial cell line (FHC). Right: quantification of the relative protein level of KIF4A in five CRC cell lines and a normal colon epithelial cell line (FHC). **b** KIF4A immunostaining in TMAs are shown. Note: top panel, magnification ×100; bottom panel, magnification ×200. **c** The distribution of the difference in staining intensities of KIF4A in colorectal carcinoma tissues compared with paired adjacent non-cancerous tissue. Note: N, paired adjacent noncancerous tissues. C, colorectal carcinoma tissues (KIF4A expression levels were significantly higher in colorectal carcinoma compared with corresponding adjacent noncancerous tissues (*P* < 0.001, the paired Wilcoxon test). **d** High KIF4A expression is associated with poorer overall cumulative survival for colorectal carcinoma patients (*P* < 0.001, log-rank test). **e** High KIF4A expression relevant to poorer disease-free cumulative survival for colorectal carcinoma patients (*P* = 0.003, log-rank test)

To investigate the role of KIF4 in CRC development, we first detected the expression of KIF4A at the protein level in five CRC cell lines using western blotting. In comparison with a normal colon epithelial cell line (FHC), KIF4A protein expression was upregulated in all five CRC cell lines (DLD1, HCT116, LoVo, SW480, and SW620) (Fig. 1a). Subsequently, we further evaluated the endogenous KIF4A expression in 492 (86.6%) of 568 CRC samples and 493 (86.8%) of 568 non-tumor tissues in the TMAs using immunohistochemistry (IHC). The remaining samples were lost due to antigen retrieval. We found that KIF4A was predominantly located in the nucleus (Fig. 1b). A paired Wilcoxon test of 454 cases, pairing the tumor tissue and its adjacent non-cancerous tissue, revealed that KIF4A protein expression was dramatically upregulated in cancerous tissue compared to adjacent non-cancerous tissue (Fig. 1c) (*P* < 0.001).

### Upregulated KIF4A expression is positively correlated with clinicopathological characteristics

To understand the clinical significance of KIF4A in CRC, Fisher’s exact test was used to examine the correlation of KIF4A expression in cancer with clinicopathological characteristics. The clinicopathological characteristics of the patients are summarized in Table 1. KIF4A expression was classified as low (immunoreactivity score (IRS): 0–6) or high (IRS: 8–12) group. We found that KIF4A protein expression was low in 26% (130/492) and high in 73.6% (362/492) of CRC tissues. High KIF4A protein expression was significant positively correlated with tumor diameter (*P* < 0.001), depth of invasion (*P* < 0.001), TNM: Tumor, Node, Metastases stage (*P* < 0.001), lymph node metastasis (*P* < 0.001), and distant metastasis (*P* = 0.034). In contrast, there was no striking association of KIF4A protein expression with age, gender, or differentiation.

**Table 1:** Relationship between KIF4A expression and clinicopathological features of CRC patients

Variables	KIF4A expression (*n* = 492 cases)		
	Low (%)	High (%)	*P* ^a^
All patients	130 (100)	362 (100)	
*Age (years)*			0.757
≤60	56 (43)	150 (41)	
>60	74 (57)	212 (59)	
*Gender*			0.918
Males	74 (57)	208 (57)	
Females	56 (43)	154 (43)	
*Depth of invasion* ^b^			<0.001
T1/T2	45 (35)	59 (16)	
T3/T4	84 (65)	303 (84)	
*Lymph node metastasis*			<0.001
N0	115(88)	198 (54)	
N1/N2/N3	15 (12)	164 (45)	
*Distant metastasis*			0.034
M0	129 (99)	343 (95)	
M1	1 (1)	19 (5)	
*TNM stage*			<0.001
I	41 (32)	23 (6.4)	
II	75 (58)	160 (44.2)	
III	14 (10)	160 (44.2)	
IV	0 (0)	19 (5.2)	
*Tumor diameter*			<0.001
≤5 cm	104 (80)	202 (56)	
>5 cm	26 (20)	160 (44)	
*Differentiation* ^c^			0.201
Poor	15 (12)	60 (17)	
Moderate/high	114 (88)	297 (83)	

^a^Two-sided Fisher’s exact tests

^b^The depth of invasion of cancer in one patient cannot be assessed

^c^The type of differentiation of cancer in six patients cannot be assessed

### KIF4A functions as an independent prognostic factor for CRC

We used Kaplan–Meier survival analysis and log-rank test to examine whether KIF4A expression was correlated with OS and disease-free cumulative survival for CRC patients. Our data suggested that CRC patients with a high level of KIF4A protein expression trended toward correlation with worse OS and disease-free cumulative survival compared with those with reduced KIF4A expression (*P* < 0.001 and *P* = 0.003, respectively, Fig. 1d, e). Meanwhile, the cumulative OS rate dropped from 84.2% in the low-KIF4A group to 63.0% in the high-KIFA group. The cumulative disease-free survival (DFS) rate fell from 82.2 to 61.2% when comparing the group with low-KIF4A expression to that with high KIF4A expression.

To further examine whether KIF4A expression was an independent prognostic factor for CRC, we used univariate and multivariate Cox regression models to confirm the prognostic value of KIF4A expression in CRC. Univariate Cox regression analysis suggested that KIF4A expression, distant metastasis, differentiation type, TNM stage, gender, and depth of invasion were the significant prognostic factors for the CRC patients’ OS and DFS (Table 2). In the multivariate Cox regression model, our data further confirmed that KIF4A expression remained an independent significant prognostic biomarker for OS (*P* = 0.003, hazard ratio (HR) = 2.12, 95% confidence interval (CI) = 1.30–3.47) and DFS (*P* = 0.007, HR = 2.60, 95% CI = 1.29–5.36) for the CRC patients after adjusting for TNM stage, differentiate type, distant metastasis, age, and gender (Table 3). Collectively, our results confirmed that KIF4A expression may serve as a potential independent prognostic factor for OS and DFS in CRC patients.

**Table 2:** Univariate Cox regression analysis of KIF4A expression and clinicopathologic variables predicting the survival of CRC patients

Variables^a^	Overall survival		Disease-specific survival	
	HR (95% CI)	*P*	HR (95% CI)	*P*
KIF4A	0.41 (0.26–0.66)	<0.001	0.39 (0.22–0.76)	0.002
Age	1.35 (0.98–1.86)	0.069	1.06 (0.50–1.25)	0.076
Gender	0.69 (0.50–0.95)	0.024	0.78 (0.66–1.10)	0.019
LNM	0.77 (0.55–1.06)	0.104	0.67 (0.44–1.02)	0.060
Distant metastasis	0.45 (0.23–0.89)	0.021	0.53 (0.21–1.37)	0.048
TNM stage	0.71 (0.52–0.98)	0.035	0.68 (0.45–1.02)	0.046
Differentiate	1.49 (1.01–2.21)	0.045	1.56 (0.34–2.92)	0.023
Tumor diameter	0.79 (0.57–1.08)	0.136	0.75 (0.49–1.20)	0.240
Depth of invasion	0.62 (0.40–0.96)	0.034	0.42 (0.22–0.82)	0.010

*HR* hazard ratio, *CI* confidence interval, *LNM* lymph node metastasis

^a^KIF4A: low vs high; age: ≤60 vs >60; gender: male vs female; LNM: N0 vs N1, N2, N3; depth of invasion: T1–T2 vs T3–T4; distant metastasis: M0 vs M1; differentiate: poor vs moderate and high; TNM stage was ranked as I–II vs III–IV; tumor diameter: ≤5 vs >5

**Table 3:** Multivariate Cox regression analysis models assessing the effects of covariates on overall and disease-free survival in CRC patients

Variables^a^	Overall survival		Disease-free survival	
	HR (95% CI)	*P*	HR (95% CI)	*P*
KIF4A	2.12 (1.30–3.47)	0.003	2.60 (1.30–5.36)	0.007
Age	0.74 (0.53–1.03)	0.075	0.65 (0.02–0.70)	0.099
Gender	1.24 (0.87–1.76)	0.241	1.01 (0.57–1.63)	0.905
Distant metastasis	2.17 (1.10–2.27)	0.026	0.71 (0.95–5.24)	0.035
TNM stage	1.48 (1.07–2.04)	0.020	1.65 (1.12–2.69)	0.042
Differentiate	0.74 (0.50–1.10)	0.136	1.24 (0.87–1.76)	0.241

*HR* hazard ratio, *CI* confidence interval

^a^KIF4A: low vs high; age: ≤60 vs >60; gender: male vs female; distant metastasis: M0 vs M1; differentiate: poor vs moderate and high; TNM stage: I–II vs III–IV

### KIF4A exerts a stimulative effect on CRC growth

**Fig. 2:**
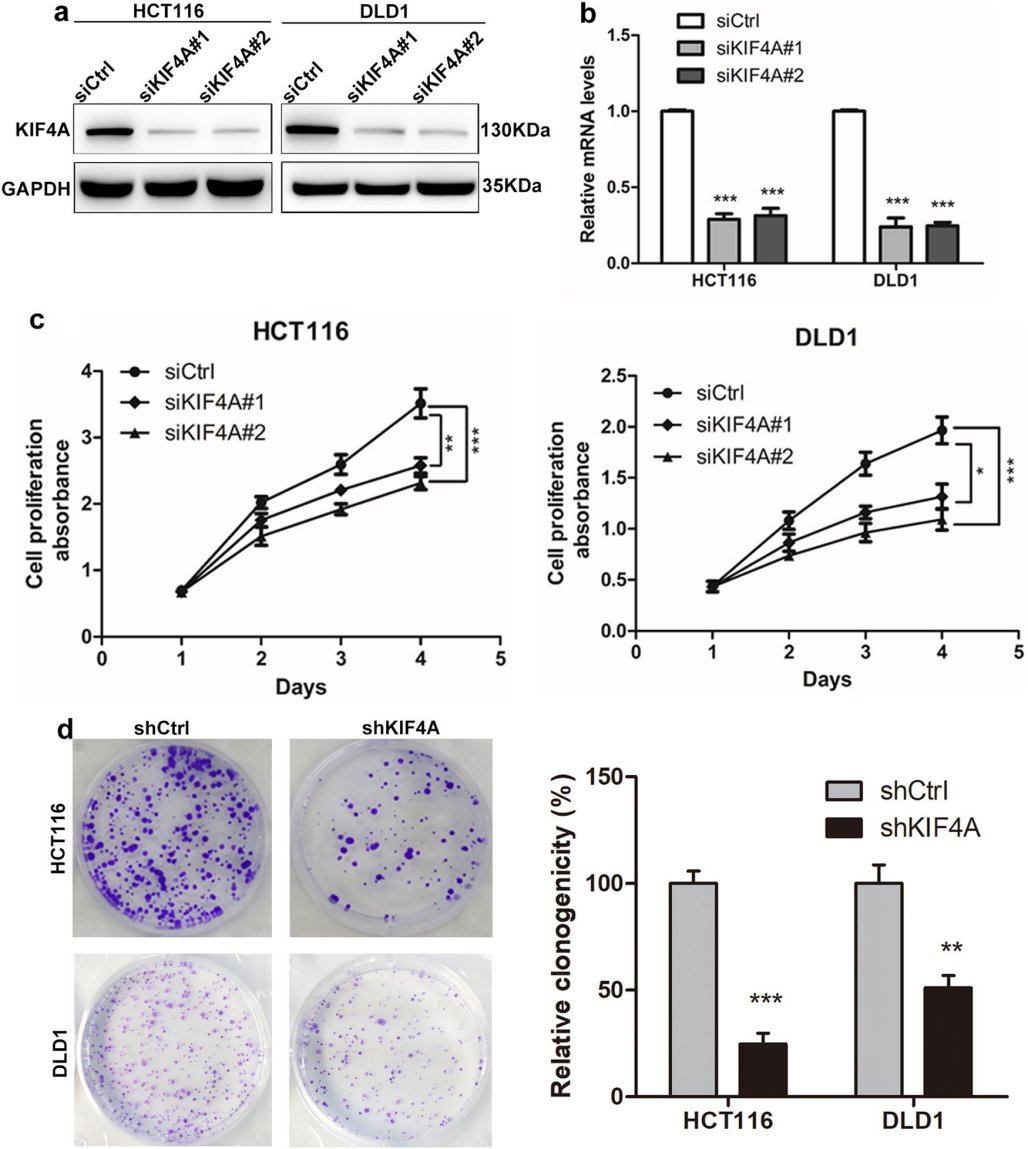
KIF4A promotes migration and invasion of CRC cells. **a** Knockdown of KIF4A was confirmed at the protein level in HCT116 and DLD1 cells by western blotting. **b** Knockdown of KIF4A was confirmed at the mRNA level in HCT116 and DLD1 cells by real-time PCR. **c** KIF4A knockdown significantly inhibited migration ability of HCT116 and DLD1 cells. **d** KIF4A knockdown distinctly suppressed invasion capacity of HCT116 and DLD1 cells. All experiments were performed in triplicate. Data are shown as mean ± standard deviations. *P < 0.05, **P < 0.01

Since our CRC cohort showed that KIF4A expression was associated with the progression of CRC, we next sought to investigate the biological function of KIF4A in CRC. HCT116 and DLD1 cells were transiently transfected with small interfering RNAs (siRNAs) targeting KIF4A (siKIF4A#1, siKIF4A#2) or control siRNA (siCtrl). Next, western blotting and quantitative real-time PCR (qRT-PCR) analysis were performed to determine the expression of KIF4A in HCT116 or DLD1 cells. The results revealed that KIF4A expression was significantly reduced in cells transfected with KIF4A siRNA compared with the control group (Fig. 2a, b). Given that the expression of KIF4A was associated with tumor diameter, we wondered whether KIF4A facilitated the proliferation of CRC cells. In a CCK-8 cell proliferation assay, we found that cell proliferation was drastically decreased in both cell lines transfected with KIF4A siRNAs compared to the control groups (Fig. 2c). The effect of KIF4A on the proliferation of CRC cells was further validated in colony formation assays. The results indicated that the cells transfected with KIF4A siRNAs exhibited a weakened capacity for colony formation compared with the control group in HCT116 cells; similar results were observed in DLD1 cells (Fig. 2d). These results indicate that KIF4A is vital for the proliferation of CRC cells.

### KIF4A facilitates the migration and invasion of colon cancer cells

**Fig. 3:**
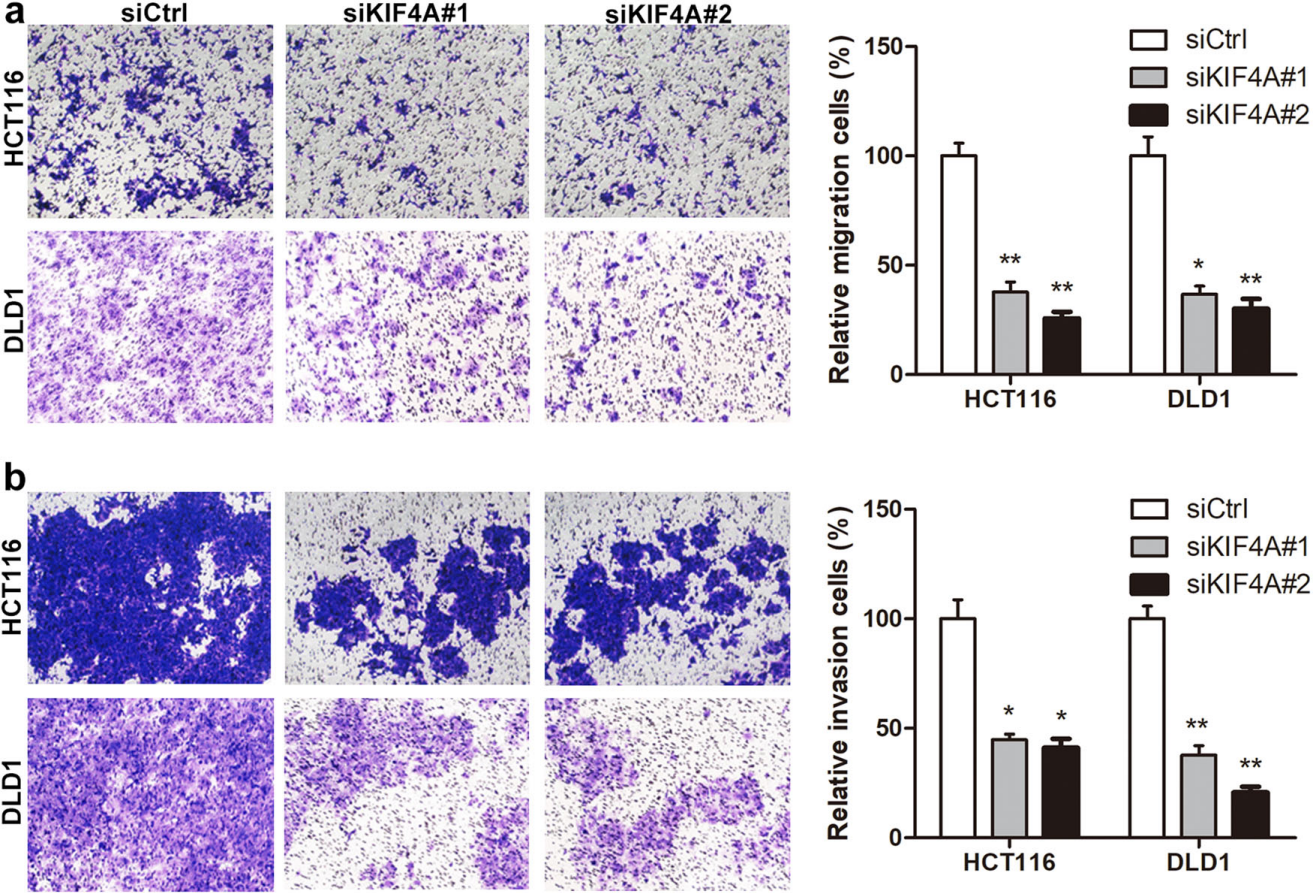
KIF4A facilitates cell proliferation and colony formation of CRC cells. **a** Knockdown of KIF4A reduces the proliferation rate of HCT116 and DLD1 cells. **b** KIF4A knockdown inhibits the capacity of colony formation in HCT116 and DLD1 cells. Data are shown as mean ± standard deviations from three independent experiments. *P < 0.05, **P < 0.01, ***P < 0.001

Considering that our clinical data indicated that high KIF4A expression is associated with cancer metastasis, we performed Transwell assays to investigate the effect of KIF4A on CRC cell migration and invasion. The results showed that downregulation of KIF4A impaired the migration ability of HCT116 and DLD1 cells compared with controls (Fig. 3a). The results of the cell invasion assay were consistent with the cell migration assay (Fig. 3b).

### Downregulation of KIF4A inhibits the G_1_/S phase transition of the cell cycle but has no effect on cell apoptosis

**Fig. 4:**
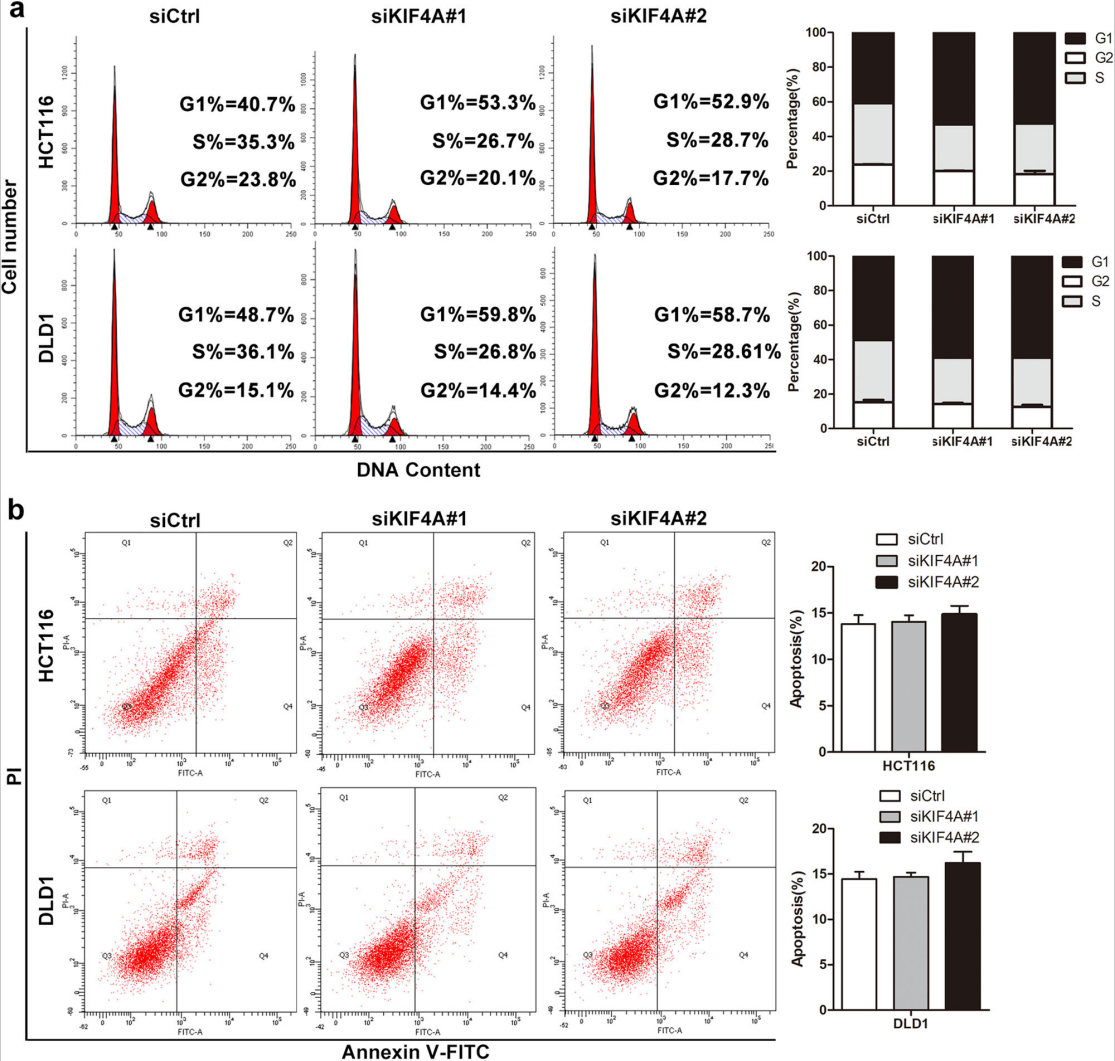
KIF4A accelerates cell cycle progression in CRC cells, but has no effect on cell apoptosis. **a** Knockdown of KIF4A increased G_0_/G_1_ phase cell population but decreased S and G_2_ phase cell population, as detected by flow cytometric analysis following Annexin FITC and PI staining. **b** Knockdown of KIF4A exerts no effect on CRC cell apoptosis, as shown by flow cytometric analysis followed by Annexin PI staining. All experiments were carried out three times independently. Data are mean ± standard deviation. *P < 0.05, **P < 0.01, ***P < 0.001

In view of our present findings, to explore the underlying mechanism by which KIF4A knockdown inhibited the proliferation of CRC cells, cell cycle analysis was performed to examine whether knockdown of KIF4A induced the inhibition of CRC cell proliferation due to arrest in a specific phase of the cell cycle. Flow cytometric analysis showed that downregulation of KIF4A markedly increased the percentage of cells in the G_0_/G_1_ phase, while the distribution of cells in S and G_2_ phase was decreased (Fig. 4a). Thus, downregulation of KIF4A seem to inhibit the G_1_/S phase transition in the cell cycle. On the other hand, because cell apoptosis also has a significant effect on cell proliferation, we next examined the influence of KIF4A on cell apoptosis using flow cytometric analysis. The results revealed that there was no effect of KIF4A on cell apoptosis (Fig. 4b). Taken together, our data demonstrate that KIF4A facilitates cell proliferation via induction of the G_1_/S phase transition.

**Fig. 5:**
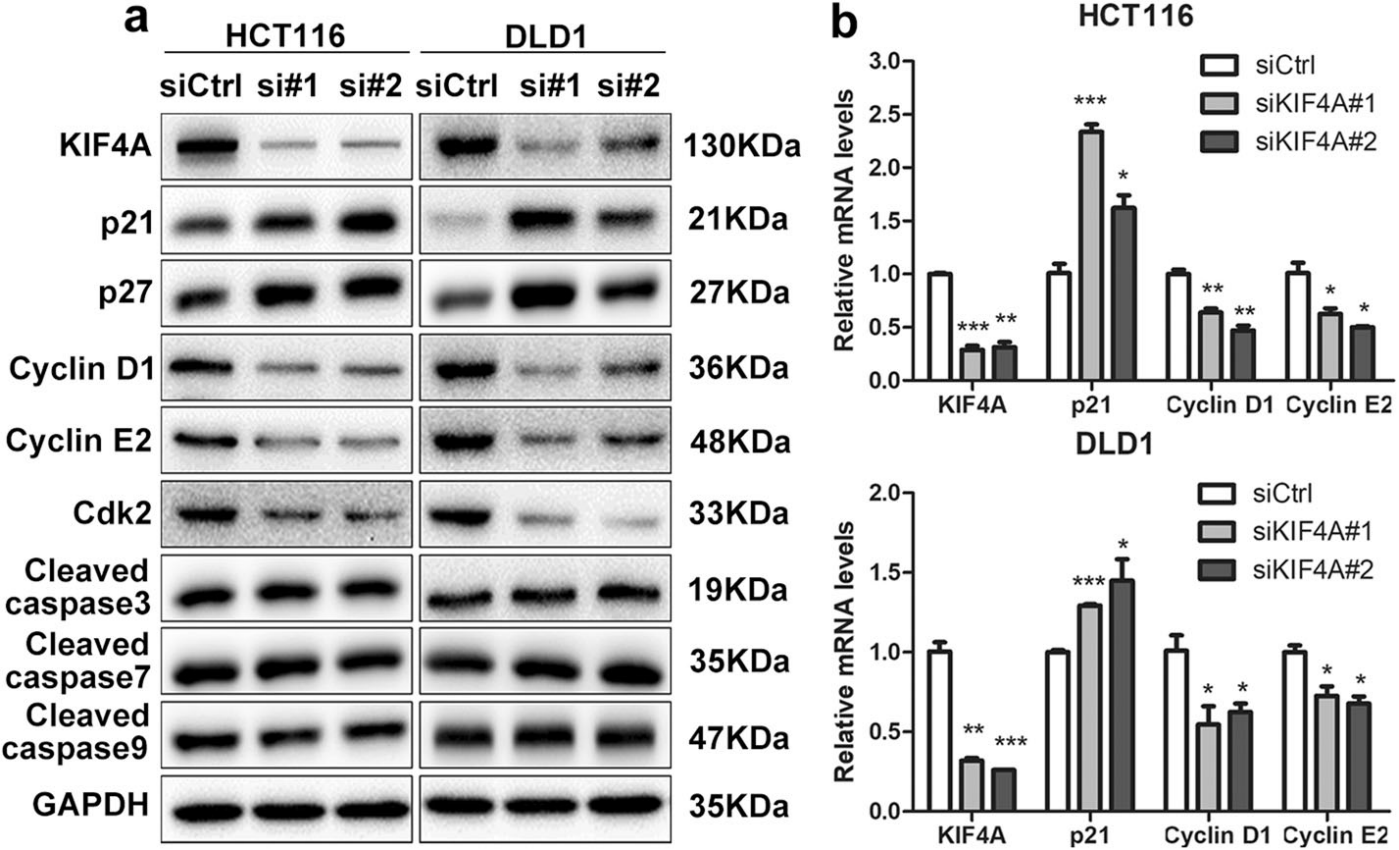
Knockdown of KIF4A induces cell cycle arrest at G_0_/G_1_ phase. **a** The expression of genes related with cell cycle and apoptosis was detected by western blotting in protein level. GAPDH was used as a reference control. **b** Relative expression of genes associated with cell cycle was detected by real-time PCR in mRNA level. GAPDH was used as a reference control. A representative experiment of three independent ones is shown. *P < 0.05, **P < 0.01, ***P < 0.001

To clarify the molecular mechanism underlying the G_1_/S phase transition, we synchronized cells at the G_0_/G_1_ phase by serum starvation for 24 h, and cells were collected 4 h after serum addition. Western blotting was performed to evaluate the expression of the key regulators associated with G_1_ phase at both the protein and messenger RNA (mRNA) level. Consistent with our flow cytometric data, the results showed that downregulation of KIF4A dramatically caused the accumulation of p21 and p27 proteins, whereas it remarkably decreased the expression of cyclin D1, cyclin E2, and cyclin-dependent kinase 2 (Cdk2) (Fig. 5a). These results were confirmed by real-time PCR, and the results indicated that p21 and p27 were negatively regulated by KIF4A at the mRNA level, while cyclin D1, cyclin E2, and Cdk2 were positively regulated (Fig. 5b). In addition, the expression of apoptosis factors including cleaved caspase 3, cleaved caspase 7, and cleaved caspase 9 was comparable (Fig. 5a).

### KIF4A transcriptionally inhibits p21 in CRC cells in vitro

**Fig. 6:**
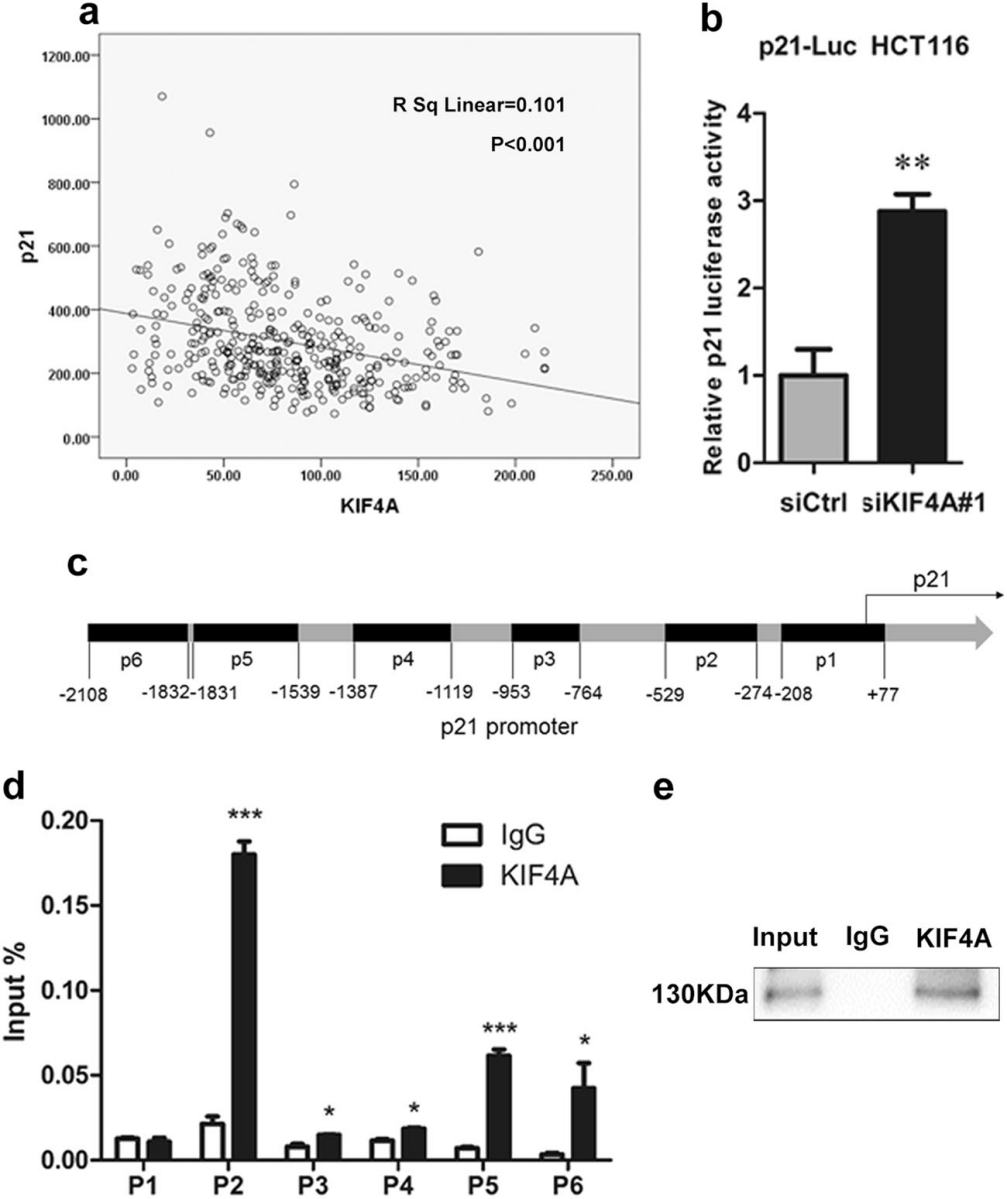
KIF4A inhibits the transcription of p21. **a** Analysis of the relationship between KIF4A expression and p21 mRNA levels in CRC patient tissues from GSE41258. **b** The p21 promoter (−2400/+11) activity was increased by KIF4A knockdown in HCT116 cells. **c** Full sequence of the human p21 promoter. p1–6 shows the regions of p21 promoter detected by the paired primers. **d** ChIP-qPCR analysis of KIF4A binding at p1, p2, p3, p4, p5, and p6 loci. **e** Western blot detection of KIF4A in the ChIP assay. Mean ± standard deviation of triplicate experiments are shown. *P < 0.05, **P < 0.01, ***P < 0.001

Cell cycle progression is regulated by the sequential activation of multiple Cdk/cyclin complexes^18^. To provide insight into the molecular mechanism underlying the G_0_/G_1_ phase cell cycle-related effect of KIF4A, we focused on the universal cell cycle inhibitor p21, a tumor suppressor involved in the regulation of G_0_/G_1_ phase arrest and cell proliferation^19^. Previous studies have demonstrated that p21 is an exceedingly regulated gene and that its gene promoter is affected by various pathways^20^. Our preceding results have confirmed that the protein and mRNA expression of p21 is increased by KIF4A knockdown; based on this, we wondered whether KIF4A participates in cell cycle progression via regulation of the p21 promoter. To verify the hypothesis, we retrieved mRNA expression data for KIF4A and p21 from 378 colorectal samples available in the Gene Expression Omnibus (GEO) database (GSE41258). Kendall Tau-b and Pearson correlation analyses demonstrated that there remains a significant negative correlation between KIF4A and p21 mRNA expression (*R*^2^ linear = 0.101, *P* < 0.001; Fig. 6a). Subsequently, a dual-luciferase reporter assay revealed that transfection of KIF4A-targeting siRNA dramatically increased the activity of the p21 promoter (Fig. 6b), suggesting a pivotal role for KIF4A in regulating the expression of the p21 at transcription level in CRC. We then searched and described the promoter region of human p21 gene (Fig. 6c) and cloned the promoter region into a luciferase reporter plasmid. Next, we analyzed the human p21 gene and designed potential binding regions in the promoter of p21; they are denoted p1, p2, p3, p4, p5, and p6 (Fig. 6c). To determine whether KIF4A could be recruited to the potential binding region in the p21 promoter, a chromatin immunoprecipitation (ChIP) assay was performed in HCT116 KIF4A knockdown and control cells. ChIP-quantitative PCR results indicated that KIF4A mainly binds to the p2-, p5-, and p6-binding regions of p21 promoter (Fig. 6d, e).

### Downregulation of p21 reverses the inhibition of proliferation, migration, and invasion induced by KIF4A knockdown

**Fig. 7:**
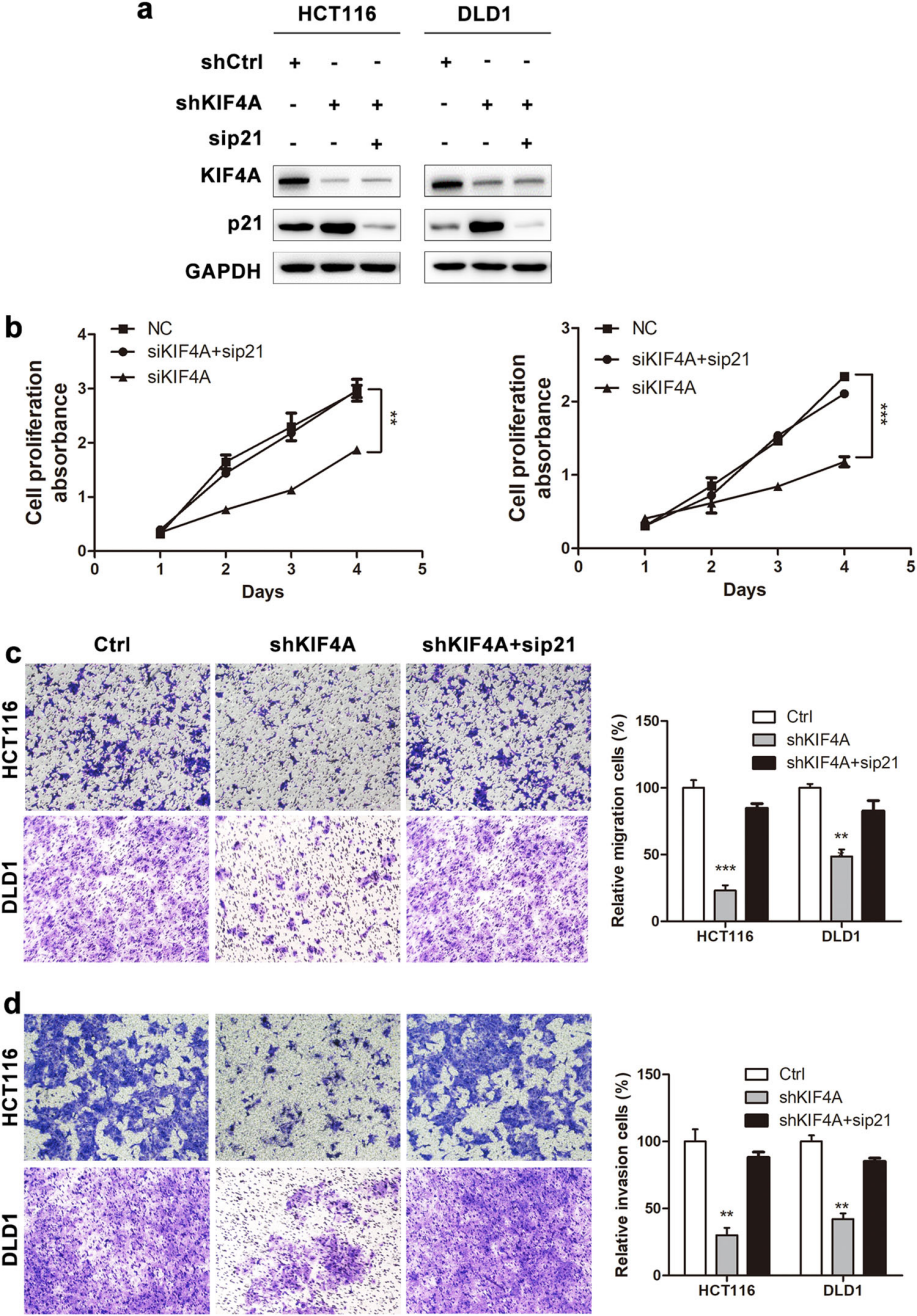
Downregulation of p21 reverses the inhibition of proliferation, migration, and invasion induced by KIF4A knockdown. **a** KIF4A and p21 knockdown in HCT116 and DLD1 stable cell line was confirmed at the protein level by western blotting. **b**–**d** The inhibition of proliferation, migration, and invasion caused by KIF4A knockdown was regressed by transient transfection of p21 siRNA in HCT116 and DLD1 stable cell lines. Data are shown as mean ± standard deviations from three independent experiments. *P < 0.05, **P < 0.01, ***P < 0.001

To assess the requirement for p21 in the inhibition of proliferation, migration, and invasion induced by KIF4A silencing, we silenced p21 by siRNA in HCT116 and DLD1 cells silencing of KIF4A (Fig. 7a). The results showed that the inhibition of proliferation, migration, and invasion caused by KIF4A knockdown was regressed by transient transfection of p21 siRNA in HCT116 and DLD1 stable cell lines (Fig. 7b–d). These results demonstrate that p21 plays a crucial role in KIF4A-regulated CRC cell proliferation, migration, and invasion.

### KIF4A promotes tumor CRC growth in vivo

**Fig. 8:**
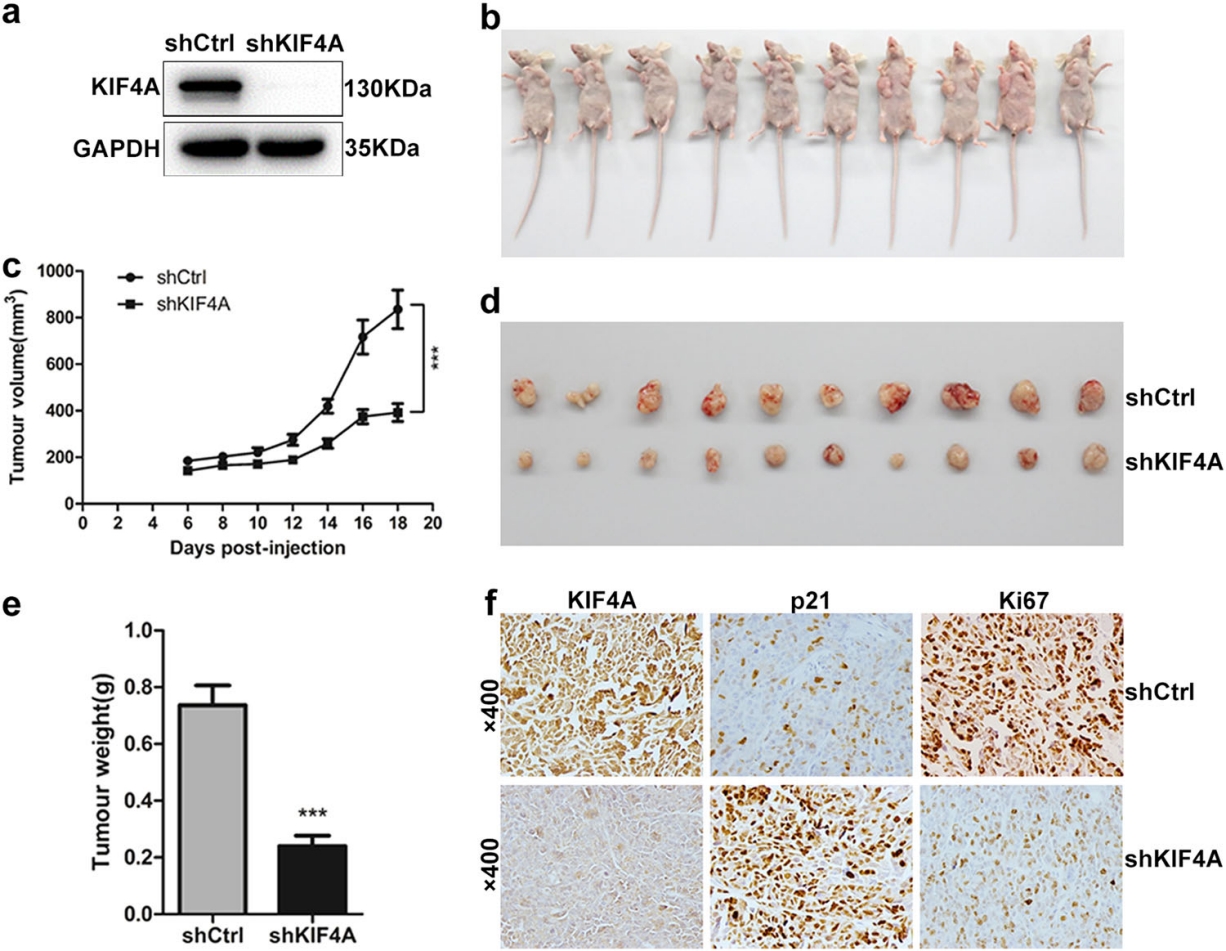
Knockdown of KIF4A restrains the tumor formation of CRC cells in vivo. **a** KIF4A knockdown in HCT116 stable cell line was confirmed at the protein level by western blotting. **b** General observation of the subcutaneous tumors in nude mice formed by HCT116 cells transfected with shKIF4A and shCtrl lentivirus (*n* = 10 in each group). **c** Tumor volumes of xenografts in nude mice. Xenografts volumes were calculated with the following formula: *V* = (*L* × *W*^2^)/2. **d** Twenty days after injection, the mice were killed and the xenograft tumors were collected. **e** The weight of the xenograft tumors was analyzed. **f** The tumor sections were performed immunochemistry staining by antibody against KIF4A, p21, and Ki67; representative images were shown (×400 magnification). ***P < 0.001. *L* the long axis, *W* the short axis

The effects of KIF4A on the proliferation of CRC cells were validated in a xenograft mouse model injected with the same number of shKIF4A or shCtrl HCT116 cells. Western blotting was used to confirm the KIF4A knockdown in HCT116 stable cell line at the protein level (Fig. 8a). The results showed that the tumors in the group injected with HCT116 cells with stable KIF4A knockdown exhibited significantly lesser tumor growth capacity (Fig. 8b) and smaller tumor volumes (Fig. 8c) than the control group. The mice were killed 20 days later, and the tumors formed by stable KIF4A knockdown cells were distinctly smaller and lighter than control group (Fig. 8d, e). Furthermore, IHC staining for tissue sections from shKIF4A and shCtrl tumors was performed to evaluate the expression of KIF4A, p21, and the nuclear cell proliferation marker Ki67. The representative images showed that knockdown of KIF4A resulted in weaker staining intensity of KIF4A and Ki67 in the excised tumors but a stronger staining intensity of p21 compared with the control group (Fig. 8f). Taken together, these results suggest that knockdown of KIF4A suppresses the proliferation of CRC cells in vivo.

## Discussion

KIF family members are involved in several cellular functions, including mitosis, meiosis, and transport of intracellular organelles and vesicles^12,21^. Given the role of KIFs in these process, dysregulation of KIFs expression and function would result in cell death, abnormality, and tumorigenesis. Several reports have demonstrated that altered KIF proteins play a crucial role in the progression of multiple human cancers^7,22,23^. As a critical member of the KIF family, KIF4A has been reported to be highly expressed and to play a role in the progression of various cancers^14,15,17,24^. However, the expression and function of KIF4A in CRC have never been investigated.

In this study, we first observed that KIF4A was located predominately in the nucleus (Fig. 1b) and was frequently highly expressed in CRC tissues when compared with paired non-tumor tissues (Fig. 1c), which agreed with previous studies in lung cancer^24^, oral squamous cell carcinomas^14^, and cervical cancer^25^. Moreover, our data demonstrated that elevated KIF4A expression was significantly correlated with clinicopathological parameters such as a deeper depth of invasion, advanced TNM stage, increased tumor diameter, increased lymph node metastasis and distant metastasis, and poorer OS and DFS (Fig. 1d, e; Table 1). In addition, univariate and multivariate Cox regression analyses revealed that high KIF4A expression was an independent adverse prognostic biomarker for CRC patients (Tables 2 and 3). These findings suggest that KIF4A plays an essential role in the progression of CRC and could function as a potential clinical prognostic predictor for CRC patients receiving curative resection.

The progression of CRC is accompanied or driven by infinite proliferation and metastasis of cancer cells^26^. Previous studies have reported that KIF4A participates in regulating the proliferation and apoptosis of cancer cells^27,28^. Our present data suggest that CRC cell proliferation and colony formation is downmodulated by blockade of KIF4A in cultured CRC cells (Fig. 2a, b). Additionally, knockdown of KIF4A distinctly led to the reduction of CRC growth in vivo (Fig. 7b, c). These findings indicate that KIF4A may contribute to the progression of CRC by promoting cell proliferation.

Accumulating evidence has demonstrated that dysregulation of the cell cycle could result in enhanced cell proliferation^29^. In addition to the inhibition of CRC cell proliferation, downregulation of KIF4A can lead to cell cycle arrest at G_0_/G_1_ phase (Fig. 4a) via the accumulation of p21 and p27 and the depletion of cyclin D1, cyclin E2, and Cdk2 at both the protein and mRNA levels (Fig. 5a, b), which are required for the G_1_ to S phase transition in cell cycle progression.

In addition, decreasing apoptosis and increasing cell proliferation is a hallmark of cancer that has been observed over the last couple of decades. Thus, a cell apoptosis assay was performed, and no evidence was found that KIF4A participates in the regulation of CRC cell apoptosis (Figs. 4b and 5a). Any disturbance of the program involved in cell cycle regulation can result in aberrant cell proliferation^30^. As a significant cell cycle regulator at the G_1_/S transition, p21, a member of the cyclin/cyclin-dependent kinase inhibitor (CKI) family, was identified as a crucial regulator for growth inhibition^20,31^. It has been confirmed that increased p21 expression acts to restrain cell proliferation and cell growth^32^. Additionally, an increasing number of studies have demonstrated that some genes are recruited to the binding sites on the p21 promoter and sequentially inhibit the transcription of p21 in various cancers^33–35^. Therefore, we wondered whether KIF4A was involved in the transcriptional regulation of p21 to promote CRC cell cycle progression at the G_1_/S transition. Consistent with previous studies, our data confirmed that elevated KIF4A can be directly recruited to the p21 promoter to inhibit its transcription in CRC cells (Fig. 6d, e). We also showed that the effect of KIF4A knockdown on PI3K/AKT and RAS/MEK/ERK signaling pathway, our data showed that downregulation of KIF4A dramatically decreased the expression of p-AKT, while the expression of RAS/MEK/ERK factors including MEK, p-MEK, ERK, and p-ERK was comparable (Supplementary Figure 1). These results indicated that KIF4A might be a regulator of PI3K/AKT signaling pathway.

Additionally, the results of the CRC charts showed that elevated KIF4A protein expression was significantly associated with increased lymph node metastasis and distant metastasis, and this effect was confirmed by transwell analysis as silencing KIF4A in CRC cells resulted in reduced cell migration and invasion capacity (Fig. 3a, b). These results indicate that the increased expression of KIF4A may play a critical role in promoting metastasis of CRC and the migration and invasion capacity of CRC cells. However, no previous studies have illustrated the precise mechanism of metastasis used by KIF4A. Metastasis of CRC is a complex progress in which angiogenesis has been reported as an essential step^36^. Restraining tumor-associated angiogenesis has been shown to be a promising tactic in preventing cancer progression and metastasis^37,38^. Several members of the KIF family have been reported to be involved in the regulation of angiogenesis. KIF26B promotes gastric cancer proliferation and metastasis by activating the VEGF pathway^23^. Overexpression of KIF14 accelerates murine retinoblastoma development through facilitating angiogenesis^39^. Based on these findings, we hypothesize that KIF4A might enhance CRC metastasis through regulating angiogenesis. On the other hand, increasing evidence has shown that cancer stem-like cells (CSCs), rather than other tumor cells, lead to cancer metastasis and recurrence^40^. Previous studies have revealed that depletion or loss of p21 results in increased self-renewal in human or mouse stem cells^35,40^. Moreover, in our study, we demonstrated that KIF4A regulated the expression of p21 via binding to its promoter region. Hence, we wondered whether KIF4A took part in CRC metastasis via p21-mediated CSC. Taken together, our results indicate that KIF4A plays a role in CRC metastasis, yet supplementary studies including in vivo and in vitro assays will help to elucidate the concrete mechanisms involved.

In conclusion, our study shows for the first time that aberrant expression of KIF4A contributes to the proliferation and progression of CRC. KIF4A facilitates cell proliferation via induction of p21-mediated cell cycle progression and promotes metastasis in colorectal cancer. These findings shed light on the prospects for KIF4A as a potential biomarker and target in prognosis and therapy for CRC.

## Materials and methods

### Patients’ specimens and construction of tissues microarrays

The tissue microarray (TMA) was  consisted of 568 cases colorectal cancer tissues and paired adjacent non-cancerous tissues, which of all were paraffin-embedded blocks and collected from the Pathology Department of Affiliated Hospital of Xuzhou Medical University. All the patients underwent radical surgery at Affiliated Hospital of Xuzhou Medical University from April 2010 to March 2015. The patients’ clinical information was gained from the Medical Record of the above-named hospital, such as clinicopathological parameters including sex, age, tumor differentiation, tumor diameter, invasion depth, lymph node metastasis, TNM stage, and others like marriage, birth place, and surgery date.

In this retrospective CRC cohort of 568 cases, there were 327 males and 241 females, and their average age was 61.7 years (range from 21 to 91). For the TNM stage, there were 349 patients at stage I and II, 219 patients at stage III and IV. For the differentiation status, 88 cases known as poorly differentiated, 391 cases were considered as moderately differentiated, and 82 tumors were well-differentiated. For the pathologic type, almost patients were deemed to be adenocarcinoma (559/568). Every patient gets a complete record of the postoperative follow-up. Survival time was calculated based on the date of surgery to the date of death or to the last follow-up. Besides, date of death was obtained from the records of the postoperative follow-up and verified by the local department of civil affairs.

The CRC TMAs was established by contract service at the National Engineering Center for Biochip, Shanghai, China, with duplicate 1.5-mm diameter cores that punched from the paraffin block, details have been previously described^41^.

### Immunohistochemistry

IHC was implemented following a standard streptavidin-peroxidase (SP) method as previously reported^42^ and heat-induced epitope retrieval was performed with the retrieval buffer, citrate, pH 6.0, prior to commencing with IHC staining protocol. For primary antibody incubation, anti-KIF4A antibodies were applied at 1:200 dilutions (ab122227, Abcam, USA), anti-p21 antibodies were applied at 1:100 dilutions (#2947, Cell Signaling Technology, USA), anti-Ki67 antibodies were applied at 1:200 dilutions (ab16667, Abcam, USA). The slide without primary antibody incubation served as negative control.

### Assessment of IHC

Two pathologists assessed separately the TMAs under blinded experimental conditions and all differences that arise were resolved by discussion. The staining scores of KIF4A were evaluated via combining the percentage of cells with the staining intensity and being dependent on the IRS. The intensity of KIF4A immunostaining was scored as 0–3 (0, negative; 1, weak; 2, moderate; 3, strong); the percentage of immunoreactivity cells was graded as 1 (0–25%), 2 (26–50%), 3 (51–75%), and 4 (76–100%). Relied on the IRS, the level of KIF4A expression was categorized as low (IRS: 0–6) and high (IRS: 8–12) expression.

### Cell lines and cell culture

The human CRC cell lines HCT116, DLD1, LoVo, SW480, and SW620 were purchased from the Shanghai Institute of Biochemistry and Cell Biology, Chinese Academy of Science (Shanghai China). HCT116, SW480, and SW620 cells were cultured in DMEM Medium while DLD1 and LoVo in RPMI 1640 medium supplemented with 10% fetal bovine serum, 100 U/ml penicillin, 100 μg/ml streptomycin, and incubated in a 37 °C humidified incubator with 5% CO_2_.

### Small interfering RNA and transient transfections

Small interfering RNA (siRNA) specific for KIF4A (siKIF4A) and non-specific control (siCtrl) were purchased from (Gene-Pharma, Shanghai, China) and transfected by siLentFect Lipid Reagent (Bio-Rad Laboratories, Inc.) according to the manufacturer’s protocol when CRC cells were grown to ~50% confluency. Six hours after transfection, the medium containing transfection reagents was replaced by fresh medium. The siRNAs sequences were described as follows:

siKIF4A#1 sense: GGUCCAGACUACUACUCUATT;

siKIF4A#2 sense: GGAAUGAGGUUGUGAUCUUTT;

sip21# sense: CCUCUGCAUUAGAAUUAUTT;

siCtrl sense: UUCUCCGAACGUGUCACGUTT.

### Stable cell line generation

For stable suppression KIF4A expression, KIF4A short hairpin RNA (shRNA) expression and control lentivirus were obtained from Gene-Pharma. HCT116 and DLD1 cells were infected with lentivirus for 48 h, and then were selected with 2 ng/ml puromycin for 2 weeks, with the medium refreshed every 3 days. The shRNA target sequences were described as follows: shKIF4A sense: GGAATGAGGTTGTGATCTT; shCtrl sense: TTCTCCGAACGTGTCACGT.

### Cell migration and invasion assay

Cell migration and invasion were carried out using modified two-chamber plates with a pore size of 8 μm. The transwell filter inserts were coated with or without Matrigel (BD Biosciences, Mississauga, Canada), respectively, for invasion and migration assay. Cells were treated with serum starvation overnight and then 2 × 10^5^ cells were seeded in the upper chamber. After incubated at 37 °C with 5% CO_2_ for 12 and 24 h, respectively, the cells that had traversed the membrane were fixed in 90% methanol and stained with crystal violet while the cells in the upper chamber were carefully removed using a cotton swab. The cells that had traversed were counted after drying.

### Cell proliferation and colony formation assay

CCK-8 analysis and colony formation assay were carried out to determine the function of KIF4A on cell proliferation. HCT116 and DLD1 cells were transfected with siRNA of KIF4A or negative control siRNA using siLentFect™ Lipid Reagentfor RNAi (Bio-Rad Laboratories, Inc.), respectively. Forty-eight hours after transfection, for CCK-8 analysis, ~4 × 10^3^ cells were seeded in each well of 96 well plates and CCK-8 solution was added 24, 48, 72, and 96 h after placing. Cells were incubated at 37 °C for 1 h after 10 μl CCK-8 solution was added. The absorbance at 450 nm was measured. For colony formation assay, 7 × 10^2^ cells were cultured in six-well plate at 37 °C for 14 days, visible colonies were washed twice with PBS, fixed, and stained with 4% paraformaldehyde and crystal violet, respectively. The number of colonies was counted visually.

### Cell cycle analysis

Forty-eight hours after transfection, the cells were synchronized by serum starvation overnight and induced re-enter cell cycle by incubating in medium containing 10% fetal bovine serum for 4 h. Then the cells were collected, washed by PBS, and fixed in pro-cooled 70% ethanol at 4 °C overnight. The day after, the cells were washed twice with PBS and resuspended in RNase A at 37 °C for 30 min, and then propidium iodide (PI) was added to the cells in the dark at 4 °C for 30 min. In the end, all samples were analyzed by flow cytometry (BD, FACSCantoTM II).

### Apoptosis

Apoptosis assay was carried out using the Annexin V-FITC/PI apoptosis detection kit (Nanjing KeyGen Biotech, Inc.) according to the manufacturer's protocol. Briefly, the cells were collected after transfection with siRNA for 48 hours, and then washed twice with PBS, and resuspended in binding buffer. Sequentially, the cells were stained with Annexin V-FITC and PI at room temperature for 15 min and then analyzed by flow cytometry (BD, FACSCantoTM II).

### Western blotting analysis

Western blotting analysis was performed as described before^43^. The specific primary antibodies against KIF4A (ab122227) and Ki67 (ab16667) was purchased from Abcam. Antibodies against p21 (#2947), p27 (#3686), cyclin D1 (#2978), cyclin E2 (#4132), Cdk2 (#2546), cleaved caspase 3 (#9661P), cleaved caspase 7 (#9491P), and cleaved caspase 9 (#9501P) were obtained from Cell Signaling Technology. Antibody against GAPDH (60004-1-Ig) was purchased from Proteintech.

### RNA extraction and quantitative real-time PCR

Total RNA was extracted from HCT116 and DLD1 using Trizol reagent (Invitrogen) according to the manufacturer’s instructions, and then the reverse transcription reaction was synthesized using the TransScript one-step guide DNA removal and complementary DNA synthesis super mix (TransGen Biotech). The sequences of all the primers amplified for qRT-PCR was listed after:

5′-CTGCAATTGGTTGGCGTCTC-3′ (forward) and 5′-CAGCGCCACTCTTACAGGAA-3′ (reverse) for KIF4A;

5′-TTTCTCTCGGCTCCCCATGT-3′ (forward) and 5′-GCTGTATATTCAGCATTGTGGG-3′ (reverse) for p21;

5′-TGAGGGACGCTTTGTCTGTC-3′ (forward) and 5′-CTTCTGCTGGAAACATGCCG-3′ (reverse) for cyclin D1;

5′-TAGCTGGTCTGGCGAGGTTT-3′ (forward) and 5′-ACAGGTGGCCAACAATTCCT-3′ (reverse) for cyclin E2;

5′-ACCAACGCAGGCGAGGGA-3′ (forward) and 5′-CCGGCTCCACAAGGAACT-3′ (reverse) for p1;

5′-GGTGTCTAGGTGCTCCAGGT-3′ (forward) and 5′-GCACTCTCCAGGAGGACACA-3′ (reverse) for p2;

5′-GCAGGAGGCAAAAGTCCTGT-3′ (forward) and 5′-GTGGTTGCAGCAGCTTTGTT-3′ (reverse) for p3;

5′-GAAAGAAGCCTGTCCTCCCC-3′ (forward) and 5′-CGCTCCCATCTACCTCACAC-3′ (reverse) for p4;

5′-CGTGGTGGTGGTGAGCTA-3′ (forward) and 5′-CTGTCTGCACCTTCGCTCCT-3′ (reverse) for p5;

5′-GTAAACCTTAGCCTGTTACTCTGAA-3′ (forward) and 5′-CATTCAATATTTCTTAAGTACCTAC-3′ (reverse) for p6;

5′-AAGGTCGGAGTCAACGGATTTG-3′ (forward) and 5′-CCATGGGTGGAATCATATTGGAA-3′ (reverse) for GAPDH.

Real-time PCR was performed using SYBR Green PCR Master Mix by a ABI-7500 qRT-PCR system thermal cycler (Vazyme Biotech, Nanjing, China). Human glyceraldehyde-3-phosphate dehydrogenase (GAPDH) relative mRNA level was served as an internal control.

### Dual-luciferase reporter assays

HCT116 cells were transiently transfected with siRNA specific for KIF4A and non-specific control siRNA using siLentFect™ Lipid Reagentfor RNAi (Bio-Rad Laboratories, Inc.), p21 promoter plasmid, renilla luciferase plasmid. Forty-eight hours post transfection, we collected the cells and measured the activities of both firefly luciferase and renilla luciferase according to the dual-luciferase reporter assay system (Promega, Madison, WI, USA). The internal standard for transfection efficiency was normalized to renilla luciferase activity. The plasmid of p21-luc (−2400/+11) was a gift from Dr. Baiqu Huang (The Institute of Genetics and Cytology, Northeast Normal University).

### Chromatin immunoprecipitation assay

ChIP assay was performed according to the protocol of ChIP assay kit (Upstate Biotechnology, Lake Placid, NY). HCT116 cells cultured in 100 mm dish (about 1 × 10^7^) were cross-linked by adding formaldehyde to final concentration of 1% and incubated in room temperature for 10 min, washed twice with cold PBS containing protease inhibitors, lysed by ChIP lysis buffer, sonicated to shear DNA at 4 °C to reduce the average length. Sonicated lysates were then diluted 10-fold with ChIP dilution buffer and reduced the non-specific binding with protein A-agarose for 1 h at 4 °C, in this step, 20 μl of lysate were taken out as input control, then followed by incubation and with anti-KIF4A or anti-IgG (as negative control) at 4 °C overnight with rotation. After reversal washes with a series of buffers, qRT-PCR was performed to amplify the genomic region of the p21 flanking the potential KIF4A-binding sites.

### Tumor xenograft study

The in vivo study was approved by the Animal Care Committee of Xuzhou Medical University. Female BALB/c nude mice (6–8 weeks old) were obtained from the Beijing Vital River Laboratory Animal Technology Co., Ltd., and maintained under specific pathogen-free conditions. HCT116 cells (5 × 10^6^) with knockdown of KIF4A and control cells were injected subcutaneously into the flanks of mice. Tumors volume (*V*) was monitored every 2 days by measuring the long axis (*L*) and the short axis (*W*) with vernier caliper and calculated with the following formula: *V* = (*L* × *W*^2^)/2. Twenty days later, the mice were killed and the tumors were weighted and processed for detecting the expression of KIF4A, p21, and Ki67 by IHC analysis.

### Statistical analysis

All the statistical analyses were performed by SPSS 20.0 statistical software package (SPSS Inc., Chicago, IL). The paired Wilcoxon test was used to assess the significance of KIF4A staining in cancers and their coupled adjacent non-cancerous tissues. The *χ*^2^ test was implemented to evaluate the relationship between KIF4A expression and clinicopathological parameters. Kendall Tau-b and Pearson correlation analyses were conducted to investigate the correlation between KIF4A and p21 expression. Probability of differences in OS as a function of time was verified by Kaplan–Meier method and log-rank test. Univariate and multivariate Cox proportional hazards regression analyses were conducted to estimate the crude HRs, adjusted HRs, and 95% CIs of HRs. *P* value <0.05 was considered as statistically significant.

## Electronic supplementary material

Supplementary figure legend

Supplementary figure 1
