# Supplementary material for: KIF4A facilitates cell proliferation via induction of p21-mediated cell cycle progression and promotes metastasis in colorectal cancer
Source: Cell Death Dis. 2018 Apr 30;9(5):477. doi: 10.1038/s41419-018-0550-9 (PMC5924760; doi:10.1038/s41419-018-0550-9)
Supplement: Supplementary file 1 — Supplementary figure legend [file 41419_2018_550_MOESM1_ESM.docx]

**Supplementary Figure 1.** **Knockdown of KIF4A repress PI3K/AKT signaling pathway.** Detection of PI3K, AKT, p-AKT, MEK, p-MEK, ERK and p-ERK by Western blots in HCT116 and DLD1 upon KIF4A knockdown.
