# Supplementary figures and images for: KIF4A facilitates cell proliferation via induction of p21-mediated cell cycle progression and promotes metastasis in colorectal cancer
Source: Cell Death Dis. 2018 Apr 30;9(5):477. doi: 10.1038/s41419-018-0550-9 (PMC5924760; doi:10.1038/s41419-018-0550-9)

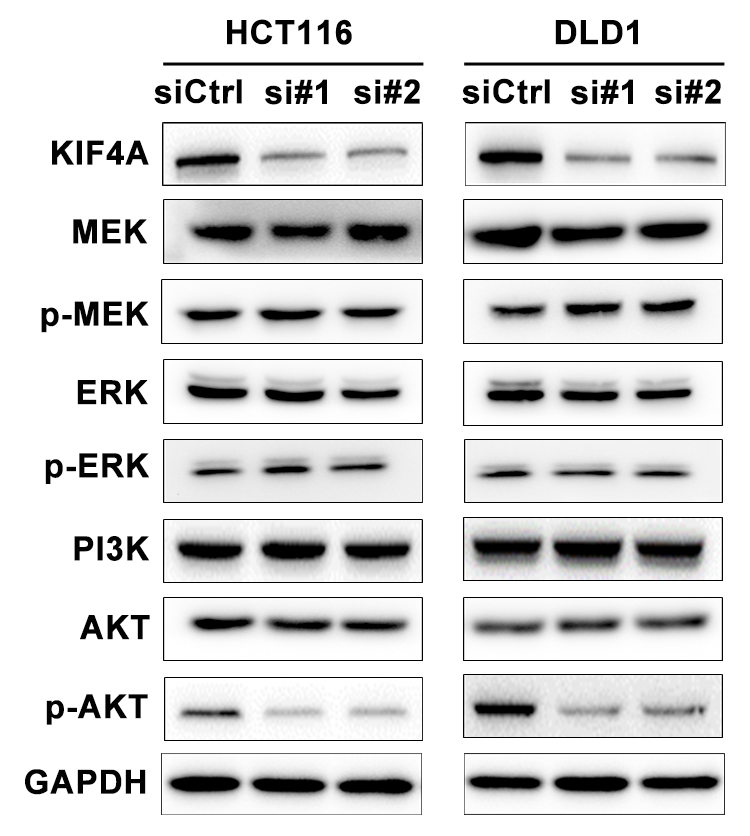

Supplement: Supplementary file 2 — Supplementary figure 1 [file 41419_2018_550_MOESM2_ESM.tif]
